# Preadmission metformin use and mortality among intensive care patients with diabetes: a cohort study

**DOI:** 10.1186/cc12886

**Published:** 2013-09-09

**Authors:** Christian Fynbo Christiansen, Martin Berg Johansen, Steffen Christensen, James M O’Brien, Else Tønnesen, Henrik Toft Sørensen

**Affiliations:** 1 Department of Clinical Epidemiology, Aarhus University Hospital, Aarhus, Denmark; 2 Department of Anesthesiology and Intensive Care, Aarhus University Hospital, Aarhus, Denmark; 3 Department of Quality and Patient Safety, Riverside Methodist Hospital, Columbus, OH, USA

## Abstract

**Introduction:**

Metformin has anti-inflammatory and anti-thrombotic effects that may improve the outcome of critical illness, but clinical data are limited. We examined the impact of preadmission metformin use on mortality among intensive care unit (ICU) patients with type 2 diabetes.

**Methods:**

We conducted this population-based cohort study among all persons admitted to the 17 ICUs in Northern Denmark (population approximately 1.8 million). We focused on all patients with type 2 diabetes who were admitted to the ICUs between January 2005 and December 2011. Through individual-level linkage of population-based medical databases, type 2 diabetes was identified using a previously validated algorithm including hospital diagnoses, filled prescriptions for anti-diabetic drugs, and elevated HbA1c levels. Metformin use was identified by filled prescriptions within 90 days before admission. Covariates included surgery, preadmission morbidity, diabetes duration, and concurrent drug use. We computed 30-day mortality and hazard ratios (HRs) of death using Cox regression adjusted for covariates, both overall and after propensity score matching.

**Results:**

We included 7,404 adult type 2 diabetes patients, representing 14.0% of 52,964 adult patients admitted to the ICUs. Among type 2 diabetes patients, 1,073 (14.5%) filled a prescription for metformin as monotherapy within 90 days before admission and 1,335 (18.0%) received metformin in combination with other anti-diabetic drugs. Thirty-day mortality was 17.6% among metformin monotherapy users, 17.9% among metformin combination therapy users, and 25.0% among metformin non-users. The adjusted HRs were 0.80 (95% confidence interval (CI): 0.69, 0.94) for metformin monotherapy users and 0.83 (95% CI: 0.71, 0.95) for metformin combination therapy users, compared to non-users. Propensity-score-matched analyses yielded the same results. The association was evident across most subgroups of medical and surgical ICU patients, but most pronounced in elderly patients and in patients with well-controlled diabetes. Former metformin use was not associated with decreased mortality.

**Conclusions:**

Preadmission metformin use was associated with reduced 30-day mortality among medical and surgical intensive care patients with type 2 diabetes.

## Introduction

Metformin is a widely used drug for treatment of type 2 diabetes [1-3] and may reduce all-cause mortality and cardiovascular event rates compared with other anti-diabetic drugs [4-6]. Beside its glucose-lowering effects [7], metformin has pleiotropic effects [8] that may be beneficial during critical illness. Experimental animal studies found that metformin has anti-inflammatory and anti-thrombotic effects that may influence the outcome of critical illness by attenuating the development and progression of acute organ dysfunction, including acute lung injury [9-11].

Only a few human studies have examined the effect of metformin in relation to critical illness. An Iranian clinical trial of 21 ICU patients detected a potentially decreased level of inflammatory cytokines when metformin was added to intensive insulin therapy [12]. In a US cohort of 1,284 diabetes patients who underwent cardiac surgery, preadmission metformin use was associated with a more than 50% decreased postoperative morbidity rate, including infections, and with a substantial decrease in inpatient mortality [13]. However, metformin may not affect mortality in patients with acute myocardial infarction [14-16], and any potential impact may be limited to conditions with severe inflammation [17,18]. As yet, no data exist on the impact of metformin on mortality after admission to an ICU.

Examination of the association between preadmission metformin use and mortality following ICU admission may improve understanding of disease processes and identify future therapeutic targets. We therefore examined whether preadmission metformin use was associated with decreased 30-day mortality among ICU patients with type 2 diabetes.

## Materials and methods

We conducted this cohort study among adults with type 2 diabetes who were admitted to an ICU in Northern Denmark (population approximately 1.8 million) between 1 January 2005 and 31 December 2011 [19]. We required that study participants had lived in the area for at least two years, in order to ensure availability of a complete history of laboratory and prescription data. Data collection was based on unambiguous individual-level linkage between population-based medical registries and databases using the unique Danish Civil Registration number assigned to each Danish citizen at birth or upon immigration [20,21]. Denmark provides tax-financed health care, with partial reimbursement of drugs, including anti-diabetic drugs, for all Danish inhabitants [22]. Northern Denmark has seventeen ICUs, including eight units in university hospitals and nine multidisciplinary units in regional hospitals. The Danish Data Protection Agency approved the study (Record No. 2009-41-3987). According to Danish law, informed consent is not required for non-interventional studies based on routinely collected data.

### Intensive care patients with type 2 diabetes

We used the Danish National Registry of Patients (DNRP) to identify adults (15 years of age or older) admitted to an ICU during the study period (n = 52,964) [19]. The DNRP contains data on virtually all admissions to Danish hospitals since 1977 and on outpatient clinic visits since 1995 [23]. Data include civil registration number, dates of hospital admission and discharge, one primary diagnosis (main reason for hospitalization), up to nineteen secondary diagnoses, surgical procedures, and major treatments. Diagnoses are coded according to the *International Classification of Diseases*, 8^th^ edition (ICD-8) until 1993 and 10^th^ edition (ICD-10) thereafter. Administration of intensive care therapy has been coded accurately since 2005 [24,25]. The DNRP is used as the data source for the Danish Intensive Care Database (a nationwide database for quality monitoring), which implies mandatory reporting and regular validation of data.

We identified type 2 diabetes using an algorithm with high validity, incorporating any previous inpatient or outpatient clinic diagnosis of diabetes after age 30 years, a diabetes diagnosis before age 30 years with no insulin prescriptions within a year before ICU admission, any filled prescription for an oral anti-diabetic drug since 1998, or a glycosylated hemoglobin A1c (HbA1c) level of 6.5% or more at the last measurement within a year before admission [19,26]. Patients with polycystic ovarian syndrome treated with metformin and no diagnosis of diabetes were excluded (n = 19) [15]. Relevant diagnostic, laboratory, and drug codes are provided in Additional file 1.

Prescription data were obtained from the Aarhus University Prescription Database, which contains data on all filled prescriptions in the study area since 1998. Data include date of dispensing, type of drug according to the anatomical therapeutic chemical (ATC) classification system, and total amount dispensed [27]. Data on HbA1c levels and other laboratory measurements were obtained from the clinical laboratory information system database, which includes results of blood tests performed at hospitals, outpatient clinics, and general practitioners [28].

### Preadmission metformin use

For each patient, we identified all prescriptions for anti-diabetic drugs, including metformin [see Additional file 1 for ATC codes]. Patients defined as current metformin users had a filled prescription within 90 days before admission; other ICU patients were defined as non-users [29]. The 90-day period was chosen because prescriptions rarely are issued for more than three months [29]. In additional analyses, we divided current users into new and long-term users [30] and considered recent (last prescription filled 91 to 365 days before ICU admission) and former use of metformin (last prescription filled 1 to 5 years before ICU admission). We included data on in-hospital administration of metformin from the day of hospital admission through the day of ICU admission, using an electronic in-hospital medication database that was implemented during the study period and available for a subset of the study population.

### Mortality

We followed patients, using the Danish Civil Registration System (DCRS), to the date of death or emigration. DCRS includes complete data on vital status, residence, and marital status for all Danish inhabitants, updated daily [20].

### Acute organ dysfunction, organ supportive treatment, and inflammation

Because any effect of metformin may be mediated through decreasing severity of organ dysfunction, we assessed acute organ dysfunction on the day of ICU admission using the laboratory cutoff values in the Sequential Organ Failure Assessment (SOFA) score criteria for kidney, liver, and coagulation system dysfunction [31]. We identified acute organ dysfunction using the laboratory database, which included a creatinine measurement on the day of ICU admission for 5,474 (73.9%) of the patients [28]. We did not include urine output to assess kidney dysfunction. For patients without a routine measurement on the day of ICU admission, we computed the mean of the values the day before and the day after this admission [31]. We also obtained data on C-reactive protein (CRP), as a marker of inflammation, and data from the DNRP on any organ supportive treatment with mechanical ventilation, renal replacement therapy, and inotropes/vasopressors.

### Potential confounders and other covariates

We used the DCRS to obtain demographic data on age, sex, and marital status as a marker of social status. We retrieved data from the DNRP on relevant inpatient and outpatient hospital contacts with a diagnosis of important preadmission chronic diseases within five years before the current admission [32]. See Additional file 1 for ICD-10 codes.

Because cardiovascular drug use may affect prognosis following intensive care, we obtained information from the prescription database on prescriptions for low-dose aspirin within 90 days, beta-blockers within 120 days, or statins within 120 days before ICU admission. These time periods reflect typical prescription durations [33-35].

In order to study possible differential impacts of metformin use in subgroups of ICU patients, we obtained data from the DNRP on diagnostic categories defined by the primary diagnosis during the current hospitalization. We defined ICU admission type as medical, acute cardiac surgical, acute non-cardiac surgical, elective cardiac surgical, and elective non-cardiac surgical according to hospital admission type and surgical procedures performed on the day of ICU admission or within seven days beforehand [36,37].

### Statistical analyses

We used contingency tables to describe covariates and rates of organ dysfunction. We followed patients from date of ICU admission until date of death, emigration, or for up to 30 days, whichever occurred first. Thirty-day mortality was assessed as one minus the Kaplan-Meier estimator. We used Cox proportional hazards regression analysis to compute hazard ratios (HRs) for death adjusted for the potential confounders in Table 1 (age, sex, marital status, preadmission morbidity, concurrent drug use, diabetes duration, and last HbA1c measurement within a year before the current admission). In an additional analysis we adjusted for organ dysfunction, although this variable may be in the causal pathway [38]. Furthermore, we compared current (new and long-term), recent, and former metformin users with never-users.

**Table 1:** Characteristics of metformin users and non-users (overall and after propensity score-matching)

	**Full cohort of all ICU patients with type 2 diabetes (n = 7,404)**				**Propensity score-matched cohort**	
	**All Metformin users**		**All Metformin users (n = 2,408), n (%)**	**Non-users (n = 4,996), n (%)**	**Metformin users (n = 2,192), n (%)**	**Non-users (n = 2,192), n (%)**
	**Metformin monotherapy users (n = 1,073), n (%)**	**Metformin combination therapy users (n = 1,335), n (%)**				
**Age group, years**						
15 to 39	23 (2.1)	21 (1.6)	44 (1.8)	141 (2.8)	43 (2.0)	42 (1.9)
40 to 59	184 (17.1)	241 (18.1)	425 (17.7)	854 (17.1)	389 (17.8)	352 (16.1)
60 to 79	731 (68.1)	939 (70.3)	1,670 (69.4)	2,965 (59.3)	1,491 (68.0)	1,505 (68.7)
80+	135 (12.6)	134 (10.0)	269 (11.2)	1,036 (20.7)	269 (12.3)	293 (13.4)
**Sex**						
Female	420 (39.1)	499 (37.4)	919 (38.2)	2,085 (41.7)	867 (39.6)	835 (38.1)
Male	653 (60.9)	836 (62.6)	1,489 (61.8)	2,911 (58.3)	1,325 (60.5)	1,357 (61.9)
**Marital status**						
Married	593 (55.3)	749 (56.1)	1,342 (55.7)	2,418 (48.4)	1,183 (54.0)	1,173 (53.5)
Never married	112 (10.4)	157 (11.8)	269 (11.2)	573 (11.5)	246 (11.2)	243 (11.1)
Divorced	154 (14.4)	179 (13.4)	333 (13.8)	769 (15.4)	311 (14.2)	286 (13.1)
Widowed	214 (19.9)	247 (18.5)	461 (19.1)	1,223 (24.5)	449 (20.5)	487 (22.2)
Unknown	0 (0.0)	3 (0.2)	3 (0.1)	13 (0.3)	3 (0.1)	3 (0.1)
**Preadmission diseases**						
Myocardial infarction	77 (7.2)	134 (10.0)	211 (8.8)	552 (11.1)	210 (9.6)	206 (9.4)
Heart failure	91 (8.5)	142 (10.6)	233 (9.7)	760 (15.2)	228 (10.4)	250 (11.4)
Peripheral vascular disease	87 (8.1)	144 (10.8)	231 (9.6)	679 (13.6)	230 (10.5)	218 (10.0)
Cerebrovascular disease	117 (10.9)	129 (9.7)	246 (10.2)	748 (15.0)	241 (11.0)	235 (10.7)
Chronic pulmonary disease	29 (2.7)	71 (5.3)	100 (4.2)	667 (13.4)	100 (4.6)	96 (4.4)
Liver disease	22 (2.1)	18 (1.4)	40 (1.7)	211 (4.2)	40 (1.8)	43 (2.0)
Moderate to severe renal disease	18 (1.7)	38 (2.9)	56 (2.3)	517 (10.4)	56 (2.6)	52 (2.4)
Cancer	140 (13.1)	149 (11.2)	289 (12.0)	695 (13.9)	277 (12.6)	269 (12.3)
Metastatic cancer	22 (2.1)	27 (2.0)	49 (2.0)	85 (1.7)	42 (1.9)	45 (2.1)
Diabetic retinopathy	44 (4.1)	142 (10.6)	186 (7.7)	452 (9.1)	182 (8.3)	167 (7.6)
Diabetic nephropathy	13 (1.2)	50 (3.8)	63 (2.6)	370 (7.4)	63 (2.9)	63 (2.9)
Hypertension	389 (36.3)	488 (36.6)	877 (36.4)	1,804 (36.1)	795 (36.3)	798 (36.4)
Clinical obesity	126 (11.7)	188 (14.1)	314 (13.0)	454 (9.1)	261 (11.9)	259 (11.8)
Alcoholism	47 (4.4)	40 (3.0)	87 (3.6)	403 (8.1)	87 (4.0)	100 (4.6)
**Diabetes duration > 5 years**	334 (31.1)	939 (70.3)	1,273 (52.9)	2,440 (48.8)	1,122 (51.2)	1,149 (52.4)
**HbA1c level**^**a**^						
< 6.50%	329 (30.7)	222 (16.6)	551 (22.9)	1,027 (20.6)	484 (22.1)	488 (22.3)
6.50% to 6.99%	214 (19.9)	168 (12.6)	382 (15.9)	1,014 (20.3)	379 (17.3)	350 (16.0)
7.00% to 7.99%	230 (21.4)	342 (25.6)	572 (23.8)	888 (17.8)	481 (21.9)	492 (22.5)
≥ 8.00%	112 (10.4)	374 (28.0)	486 (20.2)	888 (17.8)	436 (19.9)	458 (20.9)
Missing	188 (17.5)	229 (17.2)	417 (17.3)	1,179 (23.6)	412 (18.8)	404 (18.4)
**Concurrent drug use**						
Low-dose aspirin	447 (41.7)	563 (42.2)	1,010 (41.9)	1,735 (34.7)	874 (39.9)	896 (40.9)
Beta-blockers	417 (38.9)	554 (41.5)	971 (40.3)	1,888 (37.8)	878 (40.1)	895 (40.8)
Statins	674 (62.8)	898 (67.3)	1,572 (65.3)	2,203 (44.1)	1,354 (61.9)	1,387 (63.3)

^a^Last HbA1c measurement within a year before admission. Not available for the entire study area and period; n, number of patients.

We also compared metformin monotherapy users with sulfonylurea monotherapy users because they may have comparable severity of diabetes. We extended the exposure window from 90 to 180 and to 365 days to assess the sensitivity of the cut point. In addition, for the subset of patients with available in-hospital medication data, we described the proportion of patients continuing metformin use between hospital admission and ICU admission.

We also conducted analyses using propensity-score adjustment and matching, because these may be more robust when there are few outcomes per covariate [39,40]. The propensity score is the probability of being a metformin user. We estimated the propensity score for each study participant using a multivariate logistic regression model including all variables in Table 1. We adjusted for the propensity score in the Cox regression, both in an overall analysis and stratified by potential confounders, diagnostic category, admission type, and by continuation/discontinuation of metformin between hospital and ICU admission.

Finally, we carried out propensity-score matching of metformin users with non-users, which was possible in 2,192 (91.0%) patients within a range of ± 0.025. Covariates included in the estimation of the propensity score were adequately balanced after matching [41]. For the propensity-score matched analysis, we used stratified Cox regression to account for matching [42].

All analyses were conducted using the Stata software package, version 10.1. (StataCorp, College Station, TX, USA)

## Results

The study included 7,404 patients with adult type 2 diabetes, corresponding to 14.0% of 52,964 adult patients admitted to the ICUs in the study area. Among type 2 diabetes patients, 1,073 (14.5%) were metformin monotherapy users, 1,335 (18.0%) used metformin in combination with other anti-diabetic drugs, and 4,996 (67.5%) were non-users.

Descriptive data are presented in Tables 1 and 2. A larger proportion of metformin monotherapy and combination therapy users were under age 80 years, compared with non-users. Both groups of metformin users also had lower preadmission morbidity than non-users, including those with cardiovascular, liver, renal, and chronic pulmonary diseases. Diabetic nephropathy and retinopathy were more common in metformin combination therapy users and in non-users than in metformin monotherapy users. Long diabetes duration (5 years or more) and high glucose levels (HbA1c greater than 8%) within a year before admission were more common in metformin combination therapy users and less common in metformin monotherapy users, compared to non-users. Cardiovascular drugs, particularly statins, were more frequently prescribed to metformin users than to non-users (Table 1).

**Table 2:** Characteristics of current hospitalization among metformin users and non-users (overall and after propensity- score matching)

	**Full cohort of all ICU patients with type 2 diabetes (n = 7,404)**				**Propensity score-matched cohort**	
	**All Metformin users**		**All Metformin users (n=2,408)**	**Non-users (n = 4,996),**	**Metformin users (n = 2,192)**	**Non-users (n = 2,192)**
	**Metformin monotherapy users (n = 1,073)**	**Metformin combination therapy users (n = 1,335)**				
**Diagnostic category**						
Pneumonia	28 (2.6)	45 (3.4)	73 (3.0)	211 (4.2)	65 (3.0)	89 (4.1)
Septicemia	39 (3.6)	34 (2.6)	73 (3.0)	182 (3.6)	68 (3.1)	83 (3.8)
Other infectious diseases	54 (5.0)	73 (5.5)	127 (5.3)	310 (6.2)	118 (5.4)	122 (5.6)
Diabetes	11 (1.0)	33 (2.5)	44 (1.8)	170 (3.4)	42 (1.9)	59 (2.7)
Endocrinology excluding diabetes	18 (1.7)	31 (2.3)	49 (2.0)	84 (1.7)	48 (2.2)	41 (1.9)
Cardiovascular diseases	353 (32.9)	499 (37.4)	852 (35.4)	1,407 (28.2)	764 (34.9)	723 (33.0)
Respiratory diseases	75 (7.0)	107 (8.0)	182 (7.6)	357 (7.2)	164 (7.5)	156 (7.1)
Gastrointestinal and liver diseases	119 (11.1)	129 (9.7)	248 (10.3)	624 (12.5)	226 (10.3)	230 (10.5)
Cancer and other neoplasms	133 (12.4)	129 (9.7)	262 (10.9)	566 (11.3)	243 (11.1)	256 (11.7)
Trauma and poisoning	114 (10.6)	104 (7.8)	218 (9.1)	415 (8.3)	193 (8.8)	160 (7.3)
Other	129 (12.0)	151 (11.3)	280 (11.6)	670 (13.4)	261 (11.9)	273 (12.5)
**ICU admission type**						
Medical	391 (36.4)	502 (37.6)	893 (37.1)	2,090 (41.8)	829 (37.8)	853 (38.9)
Acute, non-cardiac surgery	288 (26.8)	332 (24.9)	620 (25.8)	1,476 (29.5)	568 (25.9)	592 (27.0)
Acute, cardiac surgery	43 (4.0)	52 (3.9)	95 (4.0)	174 (3.5)	86 (3.9)	88 (4.0)
Elective, non-cardiac surgery	164 (15.3)	192 (14.4)	356 (14.8)	673 (13.5)	324 (14.8)	303 (13.8)
Elective, cardiac surgery	187 (17.4)	257 (19.3)	444 (18.4)	583 (11.7)	385 (17.6)	356 (16.2)
**Biochemical evidence of organ dysfunction**^a^						
**Renal**						
Creatinine <110 μmol/L	624 (58.2)	747 (56.0)	1,371 (56.9)	2,340 (46.8)	1,222 (55.8)	1,146 (52.3)
Creatinine 110 to 299 μmol/L	222 (20.7)	310 (23.2)	532 (22.1)	1,408 (28.2)	488 (22.3)	601 (27.4)
Creatinine ≥300 μmol/L	53 (4.9)	65 (4.9)	118 (4.9)	379 (7.6)	108 (4.9)	114 (5.2)
Creatinine missing^b^	174 (16.2)	213 (16.0)	387 (16.1)	869 (17.4)	374 (17.1)	331 (15.1)
**Liver**						
Bilirubin <20 μmol/L	468 (43.6)	575 (43.1)	1,043 (43.4)	2,086 (41.8)	950 (43.3)	900 (41.1)
Bilirubin 20 to 101 μmol/L	65 (6.1)	81 (6.1)	146 (6.1)	446 (8.9)	130 (5.9)	175 (8.0)
Bilirubin ≥102 μmol/L	4 (0.4)	11 (0.8)	15 (0.6)	47 (0.9)	13 (0.6)	21 (1.0)
Bilirubin missing^b^	536 (50.0)	668 (50.0)	1,204 (50.0)	2,417 (48.4)	1,099 (50.1)	1,096 (50.0)
**Coagulation**						
Platelet count ≥150 ×10^9^/L	697 (65.0)	888 (66.5)	1,585 (65.8)	2,956 (59.2)	1,439 (65.7)	1,334 (60.9)
Platelet count 50 to 149 ×10^9^/L	187 (17.4)	228 (17.1)	415 (17.2)	938 (18.8)	366 (16.7)	443 (20.2)
Platelet count <50 ×10^9^/L	12 (1.1)	10 (0.8)	22 (0.9)	80 (1.6)	21 (1.0)	30 (1.4)
Platelet count missing^b^	177 (16.5)	209 (15.7)	386 (16.0)	1,022 (20.5)	366 (16.7)	385 (17.6)
**C-reactive protein**^a^**-**median mg/L (IQR)	103 (22-282)	112 (24-287)	107 (23-284)	179 (43-365)	108 (24-284)	157 (30-350)
**ICU treatments**						
Mechanical ventilation	492 (45.9)	647 (48.5)	1,139 (47.3)	1,962 (39.3)	1,017 (46.4)	957 (43.7)
Renal replacement therapy	66 (6.2)	83 (6.2)	149 (6.2)	319 (6.4)	134 (6.1)	113 (5.2)
Treatment with inotropes/vasopressors	361 (33.6)	456 (34.2)	817 (33.9)	1,566 (31.4)	755 (34.4)	715 (32.6)

Results presented as number of patients (%) unless otherwise stated. ^a^Laboratory data on day of ICU admission. ^b^Missing data include both unmeasured and unavailable results.

There was little difference in the primary diagnosis recorded for the current hospitalization, except for a larger proportion of metformin users admitted because of cardiovascular disease and a smaller proportion admitted with infectious disease compared with non-users (Table 2). Thirty-six percent of metformin monotherapy users and 38% of metformin combination therapy users had a non-surgical reason for ICU admission, compared to 42% of non-users. Admission after cardiac surgery was more frequent in metformin users (Table 2). Ten metformin users (0.4%) and no non-users had a primary diagnosis of lactic acidosis.

### Organ dysfunction, organ supportive treatment, and inflammation

Renal, liver, and coagulation dysfunction on the day of ICU admission, as evidenced by increased creatinine levels, increased bilirubin levels, and decreased platelet counts, were less common in both metformin monotherapy users and metformin combination therapy users, compared with non-users. However, the difference in renal dysfunction was less pronounced after propensity-score matching based on the other covariates (Table 2).

Metformin users were more frequently treated with mechanical ventilation than non-users (47% versus 39%), but there was virtually no difference in use of inotropes/vasopressors and renal replacement therapy (Table 2). When comparing the propensity score-matched cohorts, intensive care treatments were only slightly more common among metformin users (Table 2).

The median CRP at day of ICU admission was lower in metformin users compared with non-users, both in the full cohort (107 mg/L versus 179 mg/L) and in the propensity score-matched cohort (108 mg/L versus 157 mg/L) (Table 2).

### Mortality

Mortality data are presented in Table 3. Thirty-day mortality was 17.6% in metformin monotherapy users, 17.9% in metformin combination therapy users, and 25.0% in non-users. There were no major mortality differences between non-users who did not receive anti-diabetic drugs and users of sulfonylurea, insulin, or other/combination therapies (Table 3).

**Table 3:** Thirty-day mortality and hazard ratios for metformin users and non-users among type 2 diabetic patients admitted to ICUs in Northern Denmark

	**Number**	**30-day mortality, % (95% CI)**	**Crude HR (95% CI)**	**Adjusted**^**a **^**HR (95% CI)**	**Propensity score-adjusted HR (95% CI)**
**Overall analysis**	7,404				
**Metformin users**	2,408	17.7 (16.3-19.3)	0.68 (0.61-0.75)	0.82 (0.73-0.91)	0.84 (0.75-0.94)
Metformin monotherapy	1,073	17.6 (15.4-20.0)	0.67 (0.57-0.78)	0.80 (0.69-0.94)	0.82 (0.70-0.95)
Metformin combination therapy	1,335	17.9 (15.9-20.0)	0.68 (0.59-0.78)	0.83 (0.71-0.95)	0.86 (0.75-1.00)
**Metformin non-user**	4,996	25.0 (23.9-26.3)	1.00 (ref.)	1.00 (ref.)	1.00 (ref.)
- Sulfonylurea monotherapy	872	25.5 (22.7-28.5)	NA	NA	NA
- Insulin monotherapy	1,337	25.2 (22.9-27.6)	NA	NA	NA
- Other/combination	239	26.4 (21.3-32.5)	NA	NA	NA
- No pharmacotherapy	2,548	24.7 (23.1-26.4)	NA	NA	NA
**Subcohort with laboratory data**	5,474				
Metformin users	1,799	18.0 (16.3-19.9)	0.71 (0.62-0.80)	0.85 (0.75-0.97)	0.88 (0.77-1.01)
Metformin users, adjusted for admission organ dysfunction	1,799	18.0 (16.3-19.9)	0.71 (0.62-0.80)	0.88 (0.77-1.00)	NA
Metformin non-users	3,675	24.4 (23.1, 25.8)	1.00 (ref.)	1.00 (ref.)	1.00 (ref.)
**Propensity score-matched cohort**					
Metformin users	2,192	18.2 (16.7-19.9)	0.87 (0.76-1.00)	0.88 (0.75-1.02)	0.85 (0.73-1.00)
Metformin non-users	2,192	20.9 (19.3-22.7)	1.00 (Ref.)	1.00 (Ref.)	1.00 (ref.)
**Monotherapy comparison**	1,945				
Metformin monotherapy	1,073	17.6 (15.4-20.0)	0.65 (0.54-0.79)	0.90 (0.73-1.11)	0.77 (0.63-0.94)
Sulfonylurea monotherapy	872	25.5 (22.7-28.5)	1.00 (Ref.)	1.00 (Ref.)	1.00 (ref.)

^a^Adjusted for all variables in Table 1. HR, hazard ratio; ref., reference; NA, non applicable.

The mortality rate in metformin users was decreased in both monotherapy (adjusted HR (aHR) = 0.80, 95% CI 0.69, 0.94) and combination therapy users (aHR = 0.83, 95% CI 0.71, 0.95) compared to non-users, after adjustment for age, sex, marital status, diabetes duration, preadmission HbA1c level, preadmission morbidity, and concurrent cardiovascular medication (Table 3). Although adjustment for organ dysfunction upon ICU admission may attenuate the estimate with regard to any effect mediated through organ dysfunction, this had little influence on the combined estimate for metformin use in the portion of the cohort for whom we had these data (aHR including organ dysfunction = 0.88, 95% CI 0.77, 1.00 compared to aHR = 0.85, 95% CI: 0.75-0.97). The propensity score-adjusted analysis also provided virtually the same estimates as the overall analysis (Table 3).

In the propensity score-matched cohorts, 30-day mortality was 18.2% in metformin users and 20.9% in non-users, corresponding to an unadjusted HR of 0.87 (95% CI 0.76, 1.00). As expected, further adjustment for the variables originally included in the propensity score did not change the estimate (Table 3).

Among all 7,404 patients, 2,408 (32.5%) were current, 476 (6.4%) were recent, 591 (8.0%) were former, and 3,929 (53.1%) were never-users of metformin. Compared to never-use, current use was associated with a decreased mortality rate (aHR = 0.82, 95% CI 0.72, 0.92), whereas there was no clear association with recent use (aHR = 0.92, 95% CI 0.75, 1.13) and former use (aHR = 1.08, 95% CI 0.90, 1.28). Among current users, 146 were new users and 2,262 were long-term users. The decrease in mortality was similar, but imprecise, in new users (aHR = 0.76, 95% CI 0.51, 1.15) compared with long-term users (aHR = 0.82, 95% CI 0.73, 0.93).

Comparison of metformin monotherapy users (n = 1,073) with sulfonylurea monotherapy users (n = 872) showed a less pronounced association, with an aHR of 0.90 (95% CI 0.73, 1.11), but with an aHR of 0.77 (95% CI 0.63, 0.94) in the propensity score-adjusted analysis (Table 3).

Changing the anti-diabetic drug capture window from 90 to 180 or 365 days before ICU admission slightly increased the number of metformin users, but did not change the estimates considerably.

Data on inpatient medication use between hospital admission and ICU admission were available for 994 patients, including 318 preadmission metformin users and 676 metformin non-users. Among metformin users, 163 (51.3%) continued metformin upon hospitalization. The adjusted HR comparing preadmission metformin users with non-users was 0.25 (95% CI 0.13, 0.50) among patients who received metformin during hospitalization and 0.67 (95% CI 0.44, 1.01) among those who did not.

### Stratified analyses

**Figure 1:**
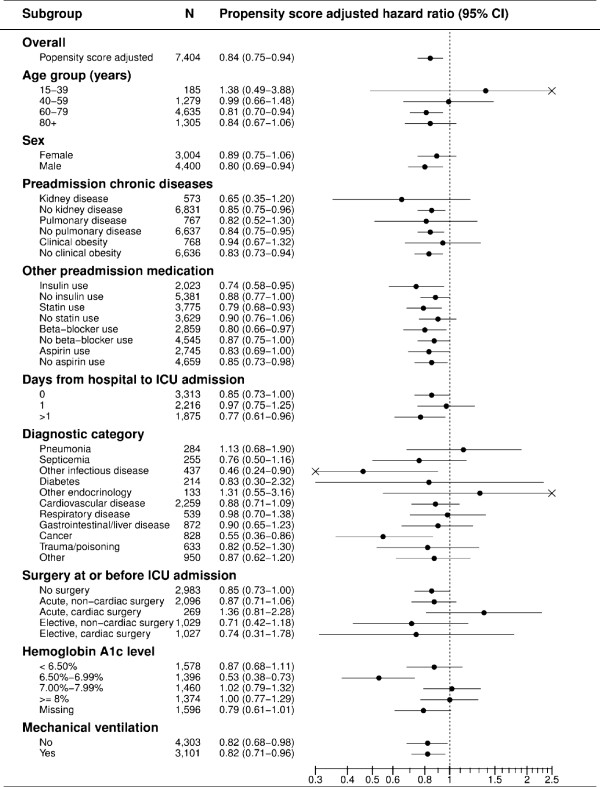
**Hazard ratios (HRs) of death within 30 days in metformin users compared with non-users. ** Adjusted by propensity score and stratified according to subgroups of type 2 diabetes patients admitted to ICUs in Northern Denmark.

Figure 1 illustrates the results of the stratified analyses. They confirm decreased mortality among metformin users across most subgroups of ICU patients, although the estimates were imprecise in several subgroups.

When stratified by patient characteristics, the decreased mortality was most pronounced in patients aged 60 years or more and in male patients. There were no major differences between patients with or without history of kidney or pulmonary disease, but the association was stronger in patients without a hospital diagnosis of obesity. The association may be more pronounced in patients using insulin or statins, which may also have immuno-modulating effects. Chronic hyperglycemia also may modulate the association, as the impact of metformin was less evident in patients with high HbA1c levels.

The potential protective effect of metformin was very similar in medical and surgical ICU patients, except for a small number of patients admitted after acute cardiac surgery. The impact of metformin use on mortality was more pronounced among patients admitted to the ICU on the day of hospital admission, compared to patients admitted the day after hospital admission. The lowered mortality in metformin users was most evident in those with a primary diagnosis of septicemia and other infectious diseases and in patients with cancer, including patients admitted with complications following cancer surgery (Figure 1).

## Discussion

This is the first study to address the association between preadmission metformin use and mortality after ICU admission. We found that users of metformin as monotherapy and in combination with other anti-diabetic drugs had decreased 30-day mortality compared to non-users. The association persisted after adjustment for preadmission morbidity and other potential confounders. Results were very similar in a propensity score-matched analysis, although the estimates were imprecise, probably because of the smaller sample size. Importantly, former use of metformin was not associated with decreased mortality.

Earlier data are very limited on metformin use and outcome of critical illness. A US cohort study of 1,284 predominantly elective cardiac surgery patients included 443 preadmission metformin users and 443 non-users in a propensity score-matched analysis. Metformin users had fewer postoperative complications, including infections (0.7% in metformin users versus 3.2% in non-users). In-hospital mortality was as low as 0.7% in metformin users compared with 1.4% in non-users (odds ratio = 0.5; 95% CI 0.1, 2.0) [13]. An Iranian randomized trial of 21 patients with systemic inflammatory response syndrome and hyperglycemia examined the effect of metformin during treatment in the ICU and found a non-significant decrease in pro-inflammatory cytokines on day 7 and a reduced insulin requirement when metformin was added to intensive insulin therapy [12]. This is supported by our finding of a lower CRP level upon admission in metformin users compared with non-users, although interpretation of their study results was hampered by the small study population, the exclusion of six patients after randomization, and by a lack of data on clinical outcomes.

Our study also supports findings from experimental animal studies that found metformin treatment to be associated with decreased mortality in lipopolysaccharide-induced acute lung injury or endotoxemia [9,11]. These effects were mediated through attenuation of the pro-inflammatory response, including a decrease in pro-inflammatory cytokines, such as TNF-α and IL-1β, and decreased neutrophil activation through mitochondrial inhibition [9,11]. The hyper-inflammatory response is a central feature of pathogenesis in the early phase of sepsis and organ dysfunction [17], and early metformin treatment may modulate this response beneficially. Beside anti-inflammatory effects, the pleiotropic properties of metformin include fibrinolytic effects that may prevent microvascular thrombosis by reducing the level of plasminogen activator [10]. We did not have clinical data to support previous animal studies that indicated a lower rate of acute lung injury [11]. In fact, we found an increased rate of mechanical ventilation in metformin users compared with non-users, but this may be explained by more metformin users being admitted after cardiac surgery.

Any effect of preadmission metformin use in our study most likely resulted from mediation of the early response to critical illness, because metformin is frequently switched to insulin upon hospital admission. This is supported by our finding of a more pronounced impact of metformin in patients who continued metformin during hospitalization and in patients admitted to the ICU on the day of hospitalization, as these patients most likely received their usual anti-diabetic drugs during the early phase of critical illness. Interestingly, we found the most pronounced effect in patients with well-controlled diabetes with a low HbA1c level, suggesting a potential interaction with preadmission glucose control or associated lifestyle factors.

Several issues should be considered in interpreting our data. We had accurate data on ICU admissions, prescription data, and death during follow up, which minimizes information bias and selection bias. We used prescriptions for anti-diabetic drugs as a proxy for current use, but any non-adherence would most likely bias our estimates towards no association. We also included patients not receiving any anti-diabetic drugs in the comparison group of non-users. This is unlikely to bias our results, as we found virtually the same mortality in this group as in other non-users, probably because this group comprised a mix of mild and non-compliant diabetes patients. While we had data on routine biochemical parameters in most patients, we lacked detailed clinical data on cardiovascular, respiratory, and cerebral dysfunction as well as urine output needed to compute the entire SOFA score or similar severity of illness scores. Still, the SOFA score upon admission may reflect chronic as well as acute organ dysfunction.

More metformin users than non-users were admitted after surgery; however, we found a potential beneficial effect in both medical and surgical ICU patients. Although we did not include data to assess the incidence of ICU admission in metformin users and non-users, we do not believe that there is any major difference as the prevalence of metformin use was very similar in our study compared with the prevalence in type 2 diabetics included in a population-based survey in 2006 [43]. Metformin is contraindicated in patients with severe congestive heart failure or with severe liver or renal disease, and should be used with caution in patients aged 80 years or older and in patients with chronic obstructive pulmonary disease [1,44,45]. We adjusted for age and diagnosed lifestyle-related conditions, such as chronic pulmonary disease, obesity, alcohol-related disease, and cardiovascular disease. Unmeasured confounding from lifestyle factors is unlikely to have a major impact on our findings because there may be no major differences in smoking, diet, and physical activity between users of various anti-diabetic drugs in Denmark [43]. However, obesity is more frequent in metformin users and may be associated with reduced mortality in ICU patients [46,47]. Results were similar for stratified analyses of patients without chronic pulmonary disease or without a diagnosis of obesity. Additionally, a true drug effect was supported by the fact that the decreased mortality was restricted to current metformin users. No association was found in former users, who are expected to be very similar with regard to the indication for prescribed metformin.

Although ICU patients may benefit from preadmission use of metformin, the effects and safety of metformin treatment initiation and continuation in patients who are already critically ill remain to be further clarified. Treatment with metformin generally is not recommended during hospitalization because of the potential risk of lactic acidosis reported in patients with severe kidney, liver, or heart disease, in patients recovering from major surgery, and in patients with shock [1,45,48]. However, we found few metformin users with a diagnosis of lactic acidosis, and the risk of this condition in metformin users may be overestimated [49]. The potential risk of lactic acidosis should be balanced against the possible benefits of metformin, and further studies are needed to assess whether routine discontinuation of metformin upon hospitalization is warranted.

In our study the non-randomized allocation of metformin treatment may have given rise to uncontrolled confounding by indication for metformin treatment, but altogether our analyses support a potential causal association between preadmission metformin use and decreased mortality. We conducted the study in a homogenous population with equal access to health care including prescription medication, which strengthened the validity of our findings. However, these findings might not be generalizable directly to other more heterogeneous health care systems.

## Conclusions

Preadmission metformin use was associated with reduced 30-day mortality among medical and surgical intensive care patients with type 2 diabetes.

## Key messages

• Metformin has anti-inflammatory and anti-thrombotic effects that may influence the outcome of critical illness, but clinical data are limited.

• We found that ICU patients who were prescribed metformin within 90 days before admission have decreased 30-day mortality compared to diabetic patients who were not preseribed metformin. Former use of metformin was not associated with decreased mortality.

• The decreased mortality was evident after adjustment for preadmission morbidity and other potential confounders and results were confirmed in a propensity score-matched analysis.

• The decreased mortality was found across most subgroups of ICU patients.

## Abbreviations

aHR: Adjusted hazard ratio; ATC: Anatomical therapeutic chemical; CRP: C-reactive protein; DCRS: Danish Civil Registration System; DNRP: Danish National Registry of Patients; HbA1c: glycosylated hemoglobin A1c; HR: Hazard ratio; ICD-10: International Classification of Diseases, 10^th^ revision; SOFA: Sequential Organ Failure Assessment.

## Competing interests

None of the authors declare any personal conflicts of interest. The Department of Clinical Epidemiology is involved in studies with funding from various companies in the form of research grants to (and administered by) Aarhus University. None of these studies have any relation to the present study.

## Authors’ contributions

HTS and CFC conceived the study idea. CFC, HTS, SC, and MBJ designed the study. MBJ and HTS collected the data. CFC and MBJ analyzed the data. CFC reviewed the literature and wrote the first draft. CFC, HTS, SC, ET, JMO, and MBJ interpreted the findings, critically reviewed and edited the manuscript, and approved the final version. All authors read and approved the final manuscript.

## Supplementary Material

Additional file 1 Codes for diagnoses, procedures, blood tests, and drugs. Click here for file
