# Supplementary material for: Preadmission metformin use and mortality among intensive care patients with diabetes: a cohort study
Source: Crit Care. 2013 Sep 9;17(5):R192. doi: 10.1186/cc12886 (PMC4057514; doi:10.1186/cc12886)
Supplement: Additional file 1 — Codes for diagnoses, procedures, blood tests, and drugs. [file cc12886-S1.pdf]

Additional file 1:

Codes for diagnoses, procedures, blood tests, and drugs

Preadmission morbidity. Diagnosis codes according to the International Classification of Diseases, 8th (ICD-8) and 10th revision (ICD-10).

	ICD-8	ICD-10
Diabetes	249, 250	E10−E14, O24 (except O24.4), G63.2, H36.0, N08.3
Myocardial infarction	*	I21, I22, I23
Congestive heart failure	*	I50, I11.0, I13.0, I13.2
Kidney disease (Moderate to severe renal disease)	*	I12, I13, N00−N05, N07, N11, N14, N18−N19, Q61
Dementia	*	F00−F03, F05.1, G30
Chronic pulmonary diseases	*	J40−J47, J60−J67, J68.4, J70.1,
		J70.3, J84.1, J92.0, J96.1, J98.2, J98.3
Connective tissue disease	*	M05, M06, M08, M09,M30,M31,
		M32, M33, M34, M35, M36, D86
Liver disease	*	B18, K70.0−K70.3, K70.9, K71, K73, K74, K76.0
		B15.0, B16.0, B16.2, B19.0, K70.4, K72, K76.6, I85
Cancer (solid tumor, leukemia, lymphoma)	*	C00−C75 (without C44)
		C91−C95
		C81−C85, C88, C90, C96
Metastatic cancer	*	C76−C80
Obesity	*	E66
Alcoholism	*	F10 (except F10.0), G31.2, G62.1, G72.1, I 42.6, K29.2, K86.0, Z72.1 (Or prescription for disulfiram. See drug code below)
Polycystic ovarian syndrome	*	E28.2
Diabetic retinopathy	*	H36.0, E10.3, E11.3, E12.3, E13.3, E14.3
Diabetic nephropathy	*	N08.3, E10.2, E11.2, E12.2, E13.2, E14.2

*Not applicable because only morbidity within five years before admission was included. (Covered by ICD-10)

Primary diagnosis during current hospitalization (diagnostic category), according to the International Classification of Diseases, 10th edition.

	**ICD-10**
Pneumonia	J12−J18, A48.1, A70.9
Septicemia	A39.2, A40, A41, A42.7, B37.7
Infectious diseases excluding pneumonia	A00−B99 (without A48.1, A70.9), G00−G07, I00−I02, I30.1, I32.0, I33, I38, I40.0, J00−J06, J36, J39.0, J10−J11, J20−J22, J85.1, J86, K35, K37, K57.0, K57.2, K57.4, K57.8, K61, K63.0, K65.0, K65.9, K67, K75.0, K75.1, K80.0, K80.3, K80.4, K81.0, K81.9, K83.0, L00−L03, L05−L08, M00, M01, M86, N10, N12, N15.1, N30, N39.0, N41, N45, N70−N77
Diabetes	E10−E14, O24 (except O24.4), G63.2, H36.0, N08.3
Endocrinology excluding diabetes	E00−E90 (without E10−E14)
Cardiovascular diseases	I00−I99 without I00−I02, I30.1, I32.0, I33, I38, I40.0
Respiratory diseases	J00−J99 without J00−J06, J36, J39.0, J10−J11, J12−J18, J20−J22, J85.1, J86
Gastrointestinal and liver disease	K00−K99 without K35, K37, K57.0, K57.2, K57.4, K57.8, K61, K63.0, K65.0, K65.9, K67, K75.0, K75.1, K80.0, K80.3, K80.4, K81.0, K81.9, K83.0
Cancer	C00−D89
Trauma and poisoning	S00−T98
Other	all codes not included in other categories
Lactic acidosis	E87.2A

**Surgical procedures**	
Surgery	All surgical codes (K-codes) in the Nordic Medico-Statistical Committee (NOMESCO) Classification of Surgical Procedures.

Drug codes according to Anatomical Therapeutic Classification (ATC).

Drug name (generic)	ATC code
All antidiabetic drugs	A10A, A10B
- Insulin	A10A
- Metformin	A10BA02
- Sulfonylureas	A10BB, A10BC
- Other antidiabetic drugs	A10B without A10BA02, A10BB, A10BC
Low-dose aspirin	B01AC06
Beta-blockers	C07
Statins	C10AA
Disulfiram	N07BB01

Blood test codes (Nomenclature for Properties and Units (NPU)-CODES and local analysis codes):

Blood test	NPU/analysis code
HbA1c	NPU03835, DNK35249, AAB00091, AAA00740, AAB00061, NPU02307, NPU27300, AAB00092
Creatinine, highest	NPU18016, NPU01807
Platelets, lowest	NPU03568
Bilirubin, highest	NPU 01370

ICU treatment codes:

Treatment	Danish treatment code
Mechanical ventilation (respirator)	BGDA0
Acute dialysis	BJFD0
Treatment with inotropes or vasopressors	BFHC92A, BFHC92B, BFHC92C, BFHC92D, BFHC92E, BFHC93A, BFHC93B, BFHC93C, BFHC95
